# Mesoporous Silica-Carbon Composites with Enhanced Conductivity: Analysis of Powder and Thin Film Forms

**DOI:** 10.3390/ma17246274

**Published:** 2024-12-22

**Authors:** Agnieszka Karczmarska, Piotr M. Zieliński, Łukasz Laskowski, Krystian Prusik, Katarzyna Pawlik, Magdalena Laskowska

**Affiliations:** 1 Institute of Nuclear Physics Polish Academy of Sciences, 31-342 Krakow, Poland; agnieszka.karczmarska@ifj.edu.pl (A.K.); pm.zielinski@ifj.edu.pl (P.M.Z.); magdalena.laskowska@ifj.edu.pl (M.L.); 2 Institute of Materials Engineering, University of Silesia in Katowice, 75 Pułku Piechoty 1A St., 41-500 Chorzów, Poland; krystian.prusik@us.edu.pl; 3 Faculty of Production Engineering and Materials Technology, Częstochowa University of Technology, 42-201 Częstochowa, Poland; katarzyna.pawlik@pcz.pl

**Keywords:** silica-carbon composite, mesoporous materials, electrical conductivity, electrical resistivity, thin film, silica SBA-15

## Abstract

The resistivity of the silica SBA-15 type can be significantly improved by forming a thin layer of carbon on the pore surface. This is possible through the carbonization reaction of a surfactant used as a structure-directing agent in the synthesis of mesostructured silica materials. The synthesis of this type of silica-carbon composite (SBA-C) is based on the use of sulfuric acid to create a carbon layer from surfactant molecules encapsulated in silica mesopores. The action of sulfuric acid takes place through dehydration and sulfonation reactions, which promote the formation of aromatic structures and favor crosslinking processes. The same procedure was applied to prepare MTF-C composites based on mesostructured thin films (MTFs). Compared to pure silica materials, these silica-carbon composites exhibit reduced pore diameter and volume while maintaining morphology and structure. The pore structure characteristics were obtained by scanning and transmission electron microscopy, X-ray energy dispersive spectroscopy, Raman spectroscopy, X-ray diffraction, thermogravimetry, and isothermal sorption analysis. The composite obtained after carbon layer formation exhibited enhanced conductivity in comparison to pure silica SBA-15. The resistivity of SBA-C composite material after annealing at 800 °C under a nitrogen atmosphere decreased to 1980 Ωcm in comparison with pure SBA-15.

## 1. Introduction

The development of novel mesoporous composites that integrate and/or enhance the properties of the individual components has become a research area of great interest among both the scientific and industrial communities [1,2,3,4]. Over the past decade, various porous carbon composites have been developed using a range of inorganic templates, including silica spheres [5,6], zeolites [7,8], alumina membranes [9,10,11], and silica sol-gels [12,13]. The undeniable driver of this heightened interest is the versatility of these composites in applications such as pollutant adsorption, catalysis, separation, electrochemistry, and gas and energy storage. In the case of the latter application, they are particularly important in batteries and supercapacitors [14,15,16,17,18]. The significance of mesostructured silica materials is due to their unique properties mainly based on their high porosity consisting of uniform mesopores (in the range of 2–10 nm) with well-defined symmetry (hexagonal, cubic or lamellar). Additionally, these materials offer the advantage of easy tuning of morphology, size, and porosity, thanks to the fact that their internal pore surfaces can be readily functionalized with a wide range of organic molecules [19,20,21,22].

Particularly noteworthy are mesoporous silica-carbon composites, based on mesoporous silica materials such as SBA-15 (Santa Barbara Amorphous No. 15), KIT-6 (Korean Institute of Technology No. 6) or MCM-41 (Mobil Composition of Matter No. 41) [23,24,25]. In these types of nanocomposites, carbon is deposited inside the pores as a thin coating, either lining the entire pore volume or part. The resulting silica-carbon composites offer numerous possibilities to create high-precision nanostructures for the control of charge transport in advanced electronic systems. One of the most prominent electronic devices utilizing materials with mesostructures is the supercapacitor [26]. These devices combine the characteristics of both capacitors and batteries, enabling them to store significant amounts of energy while allowing for rapid charge and discharge cycles, which results in high energy output in a short time [27]. For example, Hu et al. demonstrated that carbon-coated SBA-15 silica exhibits promising structural properties, including a uniform pore arrangement and high specific surface area, making it an excellent base material for electrodes in electric double-layer capacitors [28,29]. In another paper, Hu et al. reported that, when examined for its electrochemical properties, carbon-coated MCM-41 silica showed a significant specific capacitance (max 305 F/g), making it a promising candidate as a component of supercapacitors [30].

The deposition of carbon on the surface of silica pores can be carried out using three main synthetic methods: (i) chemical vapor deposition (CVD) [31,32,33,34], (ii) attachment of organic compounds to the silica surface followed by carbonization [35,36,37], and (iii) using a surfactant present during the synthesis of mesostructured silica as a structure-directing agent to create a carbon layer in the carbonization process [38,39,40]. The CVD method, although popular, often does not ensure uniform carbon coverage and requires sophisticated equipment and controlled reaction conditions. On the other hand, the method involving the attachment and carbonization of organic compounds is effective but complex, time-consuming, and requires expensive organic reagents, increasing production costs. To simplify and reduce the costs of the process, a third, more economical approach was developed. This involves the use of a surfactant (naturally used during the synthesis of mesoporous silica) that fills the pores of the silica, and its conversion to carbon during carbonization leads to the formation of a uniform carbon layer. This approach not only minimizes the number of synthesis steps but also eliminates the need for additional carbon sources or expensive reactants.

The carbonization process of the surfactant is carried out using sulfuric acid (H_2_SO_4_), which facilitates efficient dehydration and charring of the organic material. Mostly, this process is carried out at temperatures between 150 and 160 °C. The organic template serves both as a structure-directing agent and as the sole source for forming the carbon layer on the surface of the mesostructured silica. The silica-carbon composites obtained through this approach exhibit a well-defined mesoporous structure and favorable surface properties. In the case of SBA-15 and KIT-6 silicas, the three-block PEO-EO-PEO surfactant Pluronic P-123 is used, while in MCM-41 silicas, the carbon source is the cationic alkyl surfactant CTAB (cetyltrimethylammonium bromide) [38].

In view of these considerations, the aim of this work was to develop uncomplicated, efficient, and cost-effective procedures for producing mesostructured silica-carbon composites with defined conductive properties. Additionally, this study presents an analogous method for obtaining silica-carbon composites based on mesostructured thin films (MTF) obtained by the dip-coating method. At this point, it is worth noting that the structure of the MTF materials is the same as that of the SBA-15 powder material [41]. Only the form of the material itself is different (SBA-15 is in powder form and MTF is in thin film form). What is more, Marinho et al. reported that the electrical conductivity of powders is usually lower than that of the particles themselves [42]. The areas between the particles are responsible for this, providing additional resistance to the flow of charge. This means that it is extremely important and necessary to conduct this type of research on materials under compression. For this reason, following the information gathered in [43], a circuit was designed to carry out current tests.

## 2. Materials and Methods

### 2.1. Reagents

Poly(ethylene oxide)-block-poly(propylene oxide)-block-poly(ethylene oxide) copolymer (Pluronic P123, M_w_ = 5800 g/mol), tetraethyl orthosilicate (TEOS, 98%), hydrochloric acid (HCl, 33%), and sulfuric acid (H_2_SO_4_, 95.0–98.0%) were purchased from Sigma-Aldrich (St. Louis, MO, USA) and used as received.

### 2.2. Syntheses

**Figure 1:**
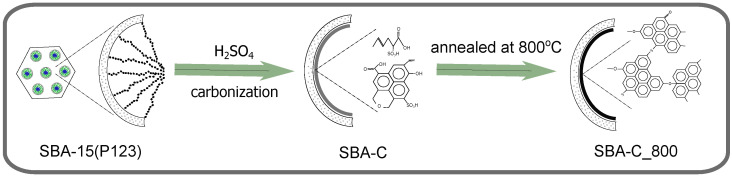
Schematic representation of the synthesis process of the mesoporous silica SBA-15 containing carbon layer inside pores. The procedure is depicted on the example of SBA-C_800; however, the schema is correct also for the MTF-C_800 material.

The synthesis process of the silica matrices used to create SBA-C and MTF-C composite materials has been described in previous works. The detailed preparation method for SBA-15 is provided in the work by El Houbbadi et al. [44], while the synthesis of mesostructured thin films (MTF) using the dip-coating method can be found in the study by Laskowski et al. [45]. The sol immersion coating process was carried out using a closed immersion chamber. Under controlled conditions of 75% relative humidity and a temperature of 22 °C inside the chamber, the substrates were withdrawn at a rate of 15 cm per minute. The coated substrates were then aged for 20 min under the same environmental conditions. Finally, all prepared thin films were subjected to overnight aging in an oven at 100 °C. As a result of this procedure, smooth thin films of SBA-15 silica with a thickness of 100 nm were obtained [45]. In both cases of silica matrix preparation, no anchor group precursor was used (the silica source was TEOS only) and the synthesis was completed after the first step, before surfactant removal (SBA-15(P123)). Thus, the matrix agent P123 in the samples was used as a carbon source. It is a three-segment copolymer (PEO)_19_-(PPO)_70_-(PEO)_19_, meaning that it consists of 19 ethylene oxide units at each end (hydrophilic segments) and 70 propylene oxide units in the middle (hydrophobic segment). In summary, Pluronic P123 provides multiple carbon-rich chains and is, therefore, a very efficient source of this material. In a further step in the synthesis of the SBA-C component, 1 g of the sample (SBA-15 silica containing P123 surfactant inside pores, hereafter named SBA-15(P123)) was immersed in 0.2 M sulfuric acid solution (approximately 10 mL of solution). The concoction was then heated at 100 °C for 6 h, then the temperature was increased to 160 °C, and the process continued for a further 12 h. The material obtained in this synthesis step is denoted as SBA-C. The final SBA-C_800 composite was obtained by thermal treatment at 800 °C for 1 h (heating rate 5 °C/min) under a nitrogen atmosphere. The procedure is depicted in Figure 1. The same procedure was followed for the MTF matrix, finally obtaining MTF-C_800.

### 2.3. Characterization

Mass changes associated with chemical reactions during sample calcination were analyzed using a thermogravimetric analyzer (TA Instruments Discovery TGA 5500, New Castle, DE, USA) under a nitrogen or oxygen atmosphere. The annealing process was carried out at a temperature of 800 °C, with a heating rate of 5 °C per minute.

The nitrogen adsorption-desorption isotherms of the samples were collected at 77.4 K by Autosorb iQ, Quantachrome Instruments (Boynton Beach, FL, USA). The Brunauer–Emmett–Teller (BET) equation and the Barret–Joyner–Halenda (BJH) method were used to evaluate the specific surface area, pore volume, and the pore size distribution (PSD) of the sample. Analysis of the microporosity was performed using a t-plot method. The calculations of specific surface area (S_BET_), pore volume, and pore size distribution were performed with ASIQWin 5.21 software. For the calculation of the BET surface area, the relative pressure range for each sample was determined by Rouquerel’s criteria [46], and seven points were always taken into account. Before analysis, the samples were degassed under vacuum conditions (approximately 10^−3^ torr) at 150 °C for 8 h.

The morphology and surface texture of the synthesized materials were examined with a Tescan Vega 3 scanning electron microscope (Brno, Czech Republic), operating at an electron beam acceleration voltage of 15 keV and a working distance of 4 mm. Additionally, X-ray energy dispersive spectroscopy (EDS) was performed using the same microscope to identify the characteristic energy lines of the elements present in the materials. For the EDS analysis, the primary beam acceleration voltage was set to 15 keV, with a working distance of 15 mm. The SEM images of thin films were taken by JEOL JSM-7100F TTLs LV/EDS field-emission scanning electron microscope (SEM) (Tokyo, Japan) operated at 15 kV acceleration voltage at the magnification 220,000×. Samples were observed as received without any sputtering.

The TEM images were collected using the FEI Tecnai G2 20 X-TWIN electron microscope (Hillsboro, OR, USA), equipped with emission source LaB_6_ and CCD camera FEI Eagle 2K.

Carbon structures were analyzed using a WITec alpha 300R confocal Raman microscope with a 532 nm laser (Oxford Instruments, Abingdon, UK). The setup included a high-performance low-dark current CCD camera (ANDOR iVac DR-316B-LDC-DD-35B, Oxford Instruments, Abingdon, UK), a Zeiss EC Epiplan-Neofluar Dic 100x/0.9 objective (Jena, Germany), and a UHTS300 SMFC VIS-NIR spectrometer (Oxford Instruments, Abingdon, UK) with a 300 mm focal length and a 600 grooves/mm grating (BLZ = 500 nm). Spectral data were collected at laser powers ranging from 0.5 to 2 mW, with 30 scans and a 1-second integration time per scan.

Small-angle X-ray diffraction (XRD) analysis was carried out on a Bruker D8 Advance diffractometer with CuKα radiation (λ = 1.5418 Å) and LynxEye detector, operated at 40 kV and 40 mA. Measurements were carried out in a conventional Bragg–Brentano setup across a 2θ range from 0.5° to 5°, with a step size of 0.01° and a measurement time of 5 s per step.

The electrical properties of all samples were assessed using a homemade, insulated Teflon cylinder with dual probes, with measurements taken at room temperature. A 10 mg sample was placed in a cylinder with a 3 mm internal diameter. The powder was compacted between two brass pistons under a force of up to 500 N, applied with a force gauge (FB500, AXIS). The powder column height was measured using a digital caliper, and resistance was recorded using an M3500A multimeter (PICOTEST). This made it possible to calculate the electrical resistivity based on equation ρ = RA/H. In this equation, the meaning of the individual symbols is as follows: R represents the electrical resistance (Ω), A is the sample’s area (m^2^), and H is the height of the powder column between the two pistons (cm).

## 3. Results

**Figure 2:**
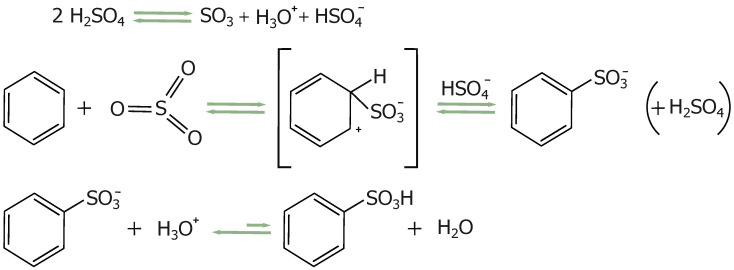
Schematic representation of the dehydration reaction and the formation of sulfonic groups.

The key role of sulfuric acid and the process of producing silica-carbon composites from mesoporous SBA-15 matrices containing the post-synthesis surfactant P123 (SBA-15(P123)) has been previously described [47]. The synthesis strategy uses surfactant molecules as a carbon source, which are employed as structure-directing agents in the synthesis of mesostructured silica. Sulfuric acid is involved in three main processes: dehydration, formation of sulfonic groups, and crosslinking (see Figure 1). The first two already occur at relatively low temperatures (160 °C) and play an important role in the formation of the carbon layer. Dehydration promotes the aromatization of the structure, while during sulfonation, sulfonic acid groups (-SO_3_H) attach to the aromatic rings in the precursor material (see Figure 2 [48]).

**Figure 3:**
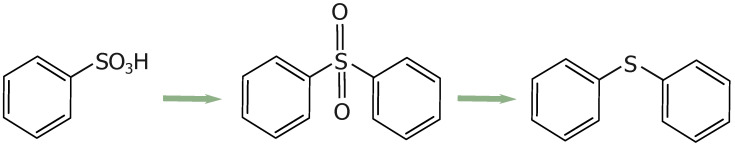
Schematic representation of the crosslinking process.

These groups increase the reactivity of the structure, especially at elevated temperatures. With a gradual increase in temperature (up to 800 °C), the sulfonic acid groups decompose, releasing water or other small molecules (SO_2_, CO_2_, CO) and facilitating the formation of sulfur-containing bridges between adjacent aromatic rings [38]. Initially, the sulfonic acid groups form sulfonyl bridges (R-SO_2_-R) between adjacent aromatic rings (R). Subsequently, some sulfonyl bridges transform into sulfide bridges (R-S-R) due to deoxidation reactions (see Figure 3) [49].

This step aids crosslinking by stabilizing the carbon framework. This mechanism is reflected in the reaction with other organic substances such as glycerol or sucrose, where the action of sulfuric acid during carbon generation appears similar to that on surfactants [38].

**Figure 4:**
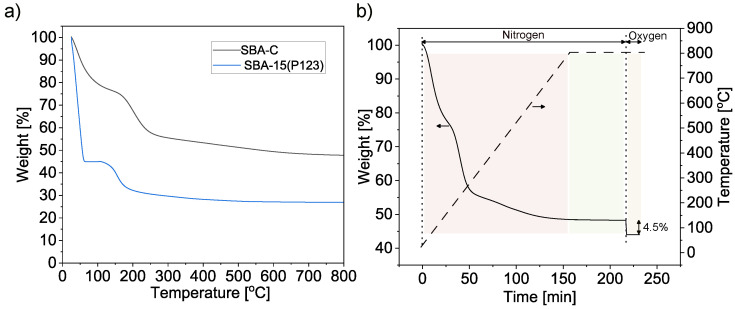
Thermogravimetric analysis of SBA-15(P123) and SBA-C composites under oxygen atmosphere (**a**) and of SBA-C composite after annealing at 800 °C for 1 h under N_2_ atmosphere and then 15 min in an oxygen atmosphere (**b**).

Thermogravimetric (TG) curves for SBA-15 (P123) and for the same material after treatment with H_2_SO_4_ acid in 160 °C and an oxygen atmosphere (SBA-C) are shown in Figure 4a. For the SBA-15(P123) material, the weight loss was 73% and is attributed to the removal of the surfactant. In the material treated with sulfuric acid, the mass decreased by about 33%. This is likely due to the elimination of a significant proportion of oxygen atoms from the P123 surfactant molecules, which were removed as H_2_O due to the dehydrating properties of the sulfuric acid [47]. Figure 4b shows the weight change of the SBA-C sample during heat treatment at 800 °C in a nitrogen atmosphere. Subsequently, oxygen was infused onto the resulting SBA-C_800 composite, leading to the oxidation of the carbon layer formed within the silica pores. This provided information about the percentage of the carbon layer (4.5% mass of sample).

**Figure 5:**
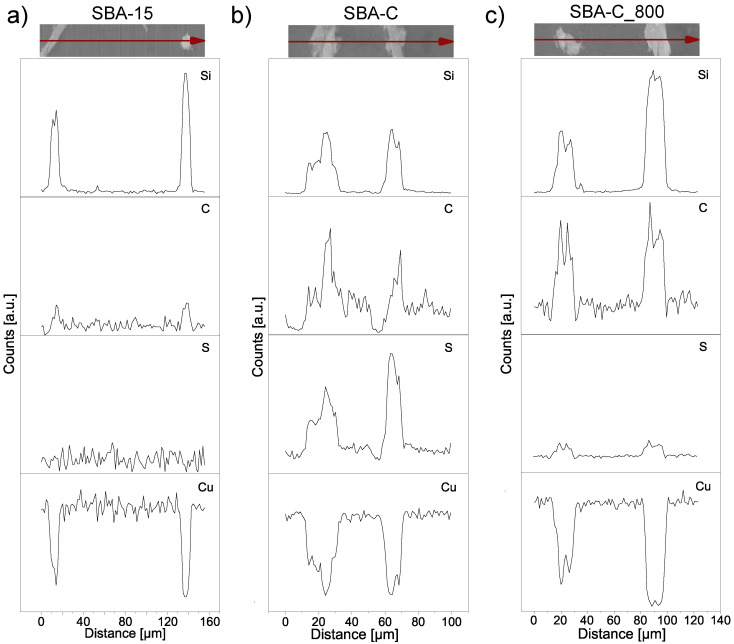
Linear EDS scan, corresponding to the SBA-15 (**a**), SBA-C composite material treated with H_2_SO_4_ up to 160 °C (**b**) and after annealing at 800 °C (**c**).

**Figure 6:**
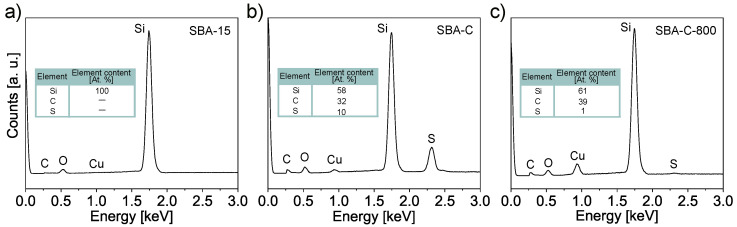
EDS spectra corresponding to the SBA-15 (**a**), SBA-C composite material treated with H_2_SO_4_ up to 160 °C (**b**) and after annealing at 800 °C (**c**). All three powders were examined on copper foil.

SBA-15(P123), SBA-C, and SBA-C_800 were also examined for their chemical composition using EDS elemental analysis, carried out during SEM observation. The results of the line scans can be seen in Figure 5. All three powders were examined on copper foil. Thanks to this, in the case of the SBA-15 sample, we only observe the share of silicon and copper (Figure 5a). In turn, for the SBA-C and SBA-C_800 complexes, the presence of carbon is observed only in places where silicon is located (i.e., in the position of the silica grain). Additionally, in Figure 5b, we observe sulfur in the place where the material occurs, which is the result of the use of sulfuric acid. After the annealing process at 800 °C in a nitrogen atmosphere, its presence was reduced nearly tenfold, as is clearly visible in Figure 5c of the EDS scan and confirmed by quantitative analysis, as shown in Figure 6c. This result confirms the occurrence of crosslinking processes within the structure via sulfur bridges. The observations conducted also verified the presence of carbon on the silica surface.

The atomic percentage carbon content of the tested SBA-C and SBA-C800 composites was more than 30%, while the sulphur content of SBA-C (non-annealed sample) was 10% (Figure 6b,c). The pure SBA-15, in which no carbon was detected, was examined as a reference (Figure 6a).

**Figure 7:**
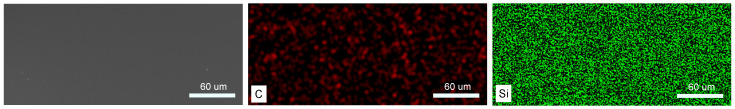
SEM image and EDS elemental scanning maps of MTF-C_800 composite.

The EDS elemental analysis was also used to characterize the MTF-C_800. Figure 7 shows the elemental mapping measurement, which also confirms the presence of C atoms in the structure of the mesostructured thin film. Blank holders do not show any signal from carbon.

**Figure 8:**
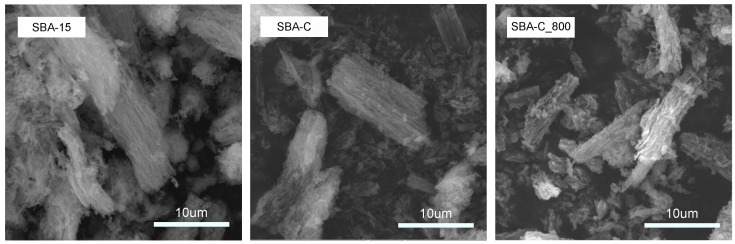
SEM images of SBA-15, SBA-C, and SBA-C_800 composites.

Scanning electron microscope images of the materials: SBA-15, SBA-C, and SBA-C_800 are shown in Figure 8. It can be clearly seen that, despite the treatment of the silica with sulfuric acid as well as high temperature (800 °C), its surface morphology did not change. For the SBA-C as well as SBA-C_800 composite materials, a morphology characterized by long, monodisperse rods, typical of SBA-15, was obtained. This demonstrates that the composite materials obtained have no structural changes other than those observed by EDS, i.e., the mesoporous surface of SBA-15 was covered by a thin layer of carbon film.

**Figure 9:**
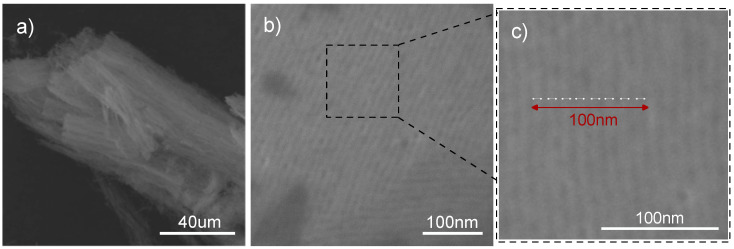
SEM images showing the MTF-C_800: after removal from the substrate (**a**), on a silicon substrate (**b**), and showing the average distance between the pores (**c**).

In the case of sample MTF-C_800, the typical morphology of the film obtained by dip-coating was also observed. Figure 9a shows a free-standing section of the film that has been removed from the substrate. A structure based on mutually adjacent homogeneous cylindrical channels can be observed. SEM imaging of the MTF-C_800 composite deposited on the substrate shows the parallel and regular alignment of the channels to its substrate (Figure 9b). An approximate pore diameter can also be estimated. In Figure 9c, the 100 nm fragment, indicated by the red bidirectional arrow, includes eight neighbouring mesopores (white bidirectional arrows), suggesting a single channel diameter of just over 10 nm.

**Figure 10:**
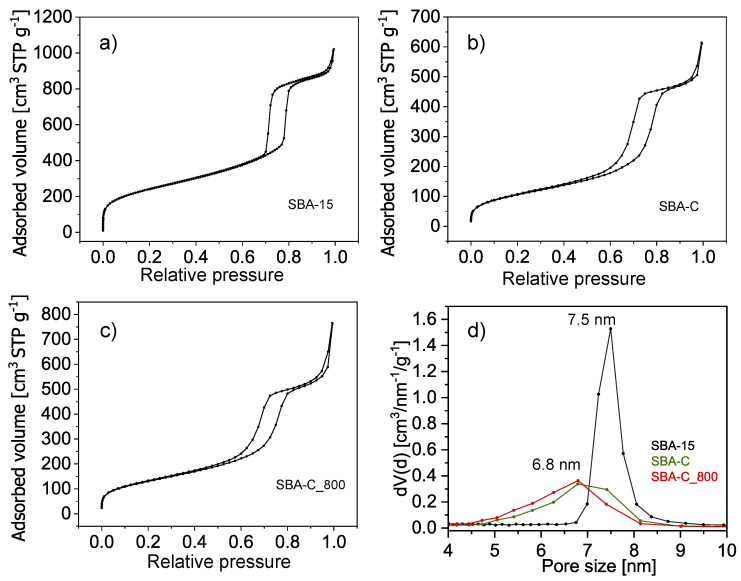
Nitrogen sorption isotherms (**a**–**c**) and pore size distributions from desorption branches (**d**) of the SBA-15, SBA-C, and SBA-C_800 composites.

To analyze the evolution of the SBA-15 porous structure during the process of carbon fabrication on the silica surface, isothermal sorption of nitrogen was applied. Only samples in powder form were analyzed due to the insufficient amount of material obtained in thin film form. Figure 10 presents three adsorption-desorption isotherms with a IV-type hysteresis loop that is typical for the SBA-15 porous structure with long uniform cylindrical mesopores arranged in a two-dimensional hexagonal lattice [50].

Table 1 summarizes the parameters calculated from the nitrogen sorption isotherms. The results show a significant decrease in the specific surface area and total pore volume of samples after being surface-coated with a layer of carbon and after annealing at 800 °C.

The specific surface area of pure silica SBA-15 after surfactant removal is 870 m^2^/g, while after carbon layer deposition on the silica surface, in the first step, it is 389 m^2^/g and 476 m^2^/g in the second step involving the process of annealing at 800 °C. The main reason for the decreased specific surface area of the silica-carbon composite in comparison to pure silica SBA-15 is the presence of a thin layer of carbon on the surface of the pores, which causes a decrease in the pore diameter from 7.5 to 6.8 nm. The creation of a carbon layer on the silica pore surface also causes blocking of micropores. The composite samples exhibit no microporosity, which in pure SBA-15 silica is responsible for 145 m^2^/g of the specific surface area.

**Figure 11:**
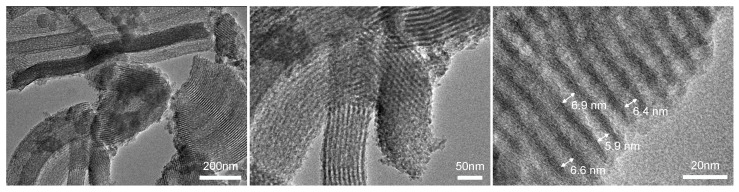
TEM images of SBA-C_800 composite.

These results are consistent with those obtained from transmission electron microscopy. Analysis of the TEM images presented in Figure 11 showed that the sample consists of a 2-D hexagonal-ordered nanotube matrix, which is the same as the structure of SBA-15 [45]. Homogeneous pore diameters ranging from 5.9 to 6.9 nm were also observed.

**Figure 12:**
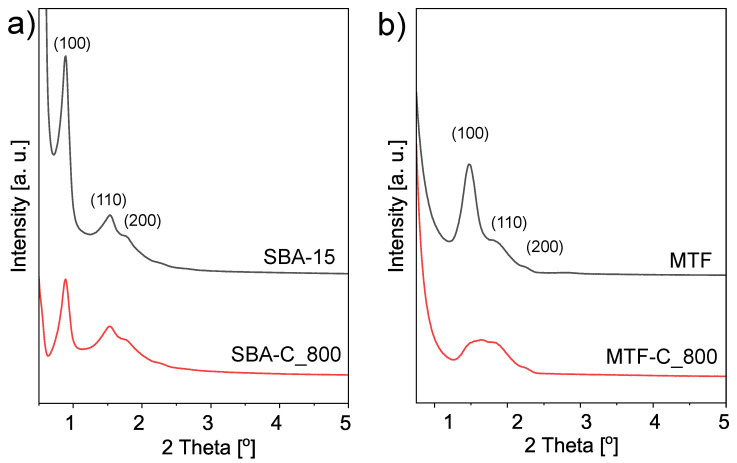
XRD patterns in the low-angle region of the mesostructured silica materials and the coresponding silica-carbon composites; powders (**a**) and thin films (**b**) forms.

The ordered structure of the SBA-C_800 composite material was also confirmed by XRD. Figure 12 shows four diffractograms of powdered samples (SBA-15 and SBA-C_800) as well as thin films (MTF and MTF-C_800). The low-angle XRD spectra exhibit clear (1 0 0), (1 1 0) and (2 0 0) reflections that indicate mesoscopic ordering. These three Bragg peaks are characteristic for 2-D hexagonally ordered lattice of silica SBA-15 and their presence in the spectra of all samples is evidence that the process of carbon layer deposition/creation did not influence the mesoscopic organization. Even annealing at 800 °C under nitrogen does not affect a highly ordered hexagonal structure. Only short-range order can be observed in the XRD data, as the reflections (110) and (200) almost disappear after annealing.

**Figure 13:**
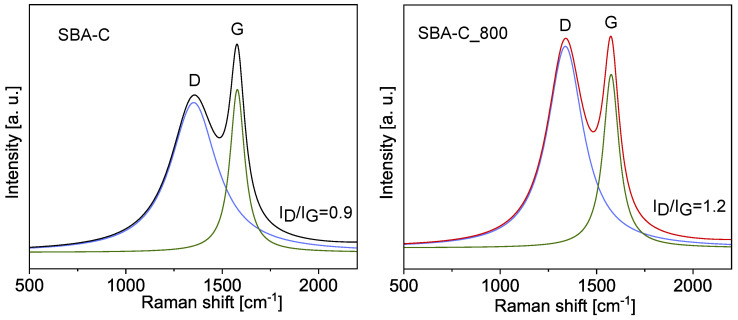
Deconvoluted Raman spectra of SBA-C and SBA-C_800 composites.

Additionally, to investigate the chemical evolution of the composite materials, including the crystalline quality and microstructure, Raman spectroscopy was employed. As shown in Figure 13, two well-defined bands are observed at approximately 1340 cm^−1^ and 1580 cm^−1^. These are referred to as the D band and the G band, respectively. The D peak corresponds to defects in carbon structures, associated with sp^3^-hybridized carbon atoms or disordered sp^2^ structures. Meanwhile, the G band is linked to C=C bond stretching in planar carbon atom structures, typical of graphite and graphene materials [51,52]. The parameter that defines the type of carbon material structure is the ratio of the D to G peak intensity (I_D_/I_G_). The fit of the spectra was analyzed using Lorentz line shapes and the parameters of these sub-peaks are shown in Table 2. The I_D_/I_G_ ratio for SBA-C was calculated to be 0.9 and increased to 1.2, which means more defects were produced after treatment at 800 °C.

**Figure 14:**
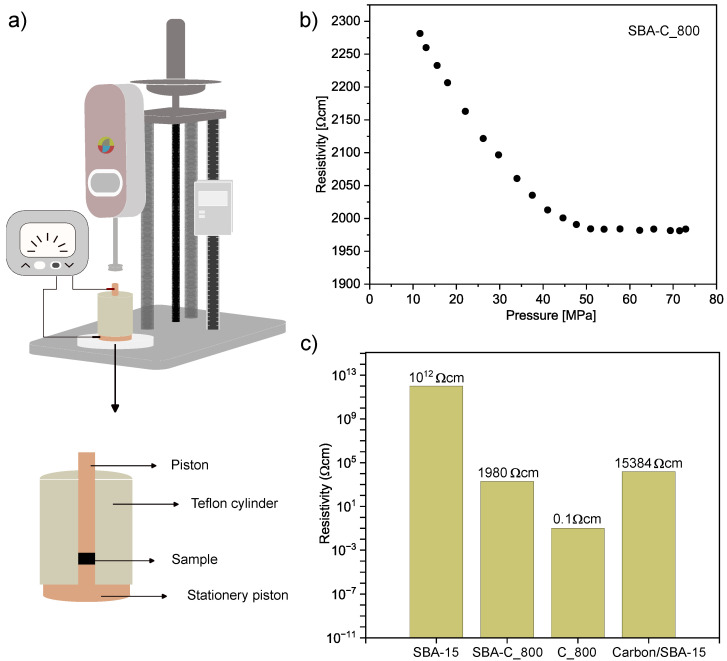
Schematic diagram of the experimental equipment for measuring electrical resistivity (**a**), electrical resistivity of SBA-C_800 as a function of pressure (**b**), electrical resistivity of SBA-15, SBA-15_800, C_800 and carbon/SBA-15 composite annealed at 800 °C [54] (**c**).

The designed current test circuit is schematically shown in Figure 14a and described in detail in the Characteristics section. Figure 14b shows the electrical behavior of the SBA-C_800 under compression in the pressure range from 10 to 72 MPa. The sample exhibits a significant drop in electrical resistance with increasing pressure. It is a well-known behavior and is attributed to the rise in the number of electrical contacts between particles under compression [53]. After exceeding 50 MPa, the resistivity value stabilizes at around 1980 Ωcm. For comparison, the resistivity of SBA-15 and pure carbon material was also measured. The measurements were carried out under the same conditions, i.e., for the same amount of powder and at the same pressure of 70 MPa. The results are shown in Figure 14c. As expected, SBA-15 displayed very high resistivity (i.e., no conductivity), while pure carbon material heated to 800 °C (C_800) had a resistivity of 0.1 Ωcm. This result partially explains the high resistance measured for SBA-C_800 (1980 Ωcm). This is probably due to the fact that the carbon coating is only formed inside the pores of the silica particles, thus causing a high inter-particle resistance. However, this is much lower than that measured in the work of Nishihara et al. [54]. They investigated a carbon/SBA-15 composite material produced by coating the pore surface of mesoporous silica with an extremely thin layer of carbon, which consisted of only 1–2 graphene sheets. Such a composite was obtained by a dehydration reaction between the surface silanol groups in SBA-15 and the hydroxyl groups of the 2,3-dihydroxynaphthalene molecules. The composite showed an electrical resistivity of about 15 kΩcm after firing also at 800 °C and at a pressure of 13 MPa. In summary, the result we obtained confirms that our material is a composite that has acquired the characteristics of a conductive material through the carbonization process.

## 4. Conclusions

We synthesized mesoporous silica-carbon composites in thin film and powder forms by direct carbonization of the surfactant P123 used as a structure-directing agent in the synthesis of mesostructured silica materials. The process was monitored by thermogravimetry, which made it possible to accurately determine the mass loss in the various stages of thermal processing of the material and to determine the amount of individual components in the resulting composite. This study was complemented by EDS studies, which, in combination with TGA, allowed the percentage of individual elements in the composites to be determined at the various stages of processing.

The structural changes occurring in the composites at the different stages of thermal treatment were analyzed in detail. After annealing at 800 °C, the studied composites retained their surface morphology and ordered mesoporous structure, which was directly confirmed by SEM and TEM observation. In addition, XRD examination showed that the material retained its ordered structure, which was slightly deformed as a result of the heat treatment and the filling of the pores with carbon.

Extending the above studies, we measured nitrogen sorption and desorption. As shown by isotherm analyses, when the silica pores were filled with a thin layer of carbon, a reduction in pores from 7.5 nm to 6.8 nm could be observed, as well as the disappearance of microporosity.

We also measured and compared with literature values the electrical properties under compression of our composites subjected to annealing at 800 °C. The resulting resistivity was 1980 Ωcm, which is many times lower than the values obtained for non-carbonised SBA-15 silica. This result demonstrates the achievement of a presumed composite, containing conductive carbon in its composition.

## Figures and Tables

**Table 1:** Structural properties of the SBA-15, SBA-C, and SBA-C_800 composites.

Sample	S_BET_ [m^2^/g]	Pore Volume [m^3^/g]	Micropore Volume [m^3^/g]	Micropore Area [m^2^/g]	Pore Diameter [nm]
SBA-15	870	1.48	0.065	162.631	7.5
SBA-C	389	0.964	0.002	17.706	6.8
SBA-C_800	476	1.176	0.001	16.393	6.8

**Table 2:** Raman factors for SBA-C and SBA-C_800 composites.

Sample	D Band (cm^−1^)		G Band (cm^−1^)		I_*D*_/I_*G*_
	$\nu_{D}$	$\omega_{D}$	$\nu_{G}$	$\omega_{G}$	
SBA-C	1352	291	1578	92	0.9
SBA-C_800	1338	226	1575	100	1.2

## Data Availability

The original contributions presented in the study are included in the article, further inquiries can be directed to the corresponding author.

## Abbreviations

The following abbreviations are used in this manuscript:

BET	Brunauer–Emmett–Teller method (equation)
BJH	Barret–Joyner–Halenda method
BLZ	Blaze wavelength
CCD	Charge-coupled device
CTAB	Cetrimonium bromide
CVD	Chemical vapor deposition
EDS	X-ray energy-dispersive spectroscopy
HCl	Hydrochloric acid
H_2_SO_4_	Sulfuric acid
KIT-6	Korean Institute of Technology No. 6
MCM-41	Mobil Composition of Matter No. 41
MTF	Mesostructured (silica) thin film
MTF-C	Silica-carbon thin film composite
MTF-C_800	Silica-carbon thin film composite heated at 800 °C
M_w_	Molecular weight
P123	Pluronic P123
SBA-15	Santa Barbara Amorphous No. 15
SBA-15(P123)	SBA-15 with surfactant P123
S_BET_	BET specific surface area
SEM	Scanning electron microscope
Si-C	Silica-carbon composite
Si-C_800	Silica-carbon composite heated at 800 °C
STP	Standard temperature and pressure
TEM	Transmission electron microscope
TEOS	Tetraethyl orthosilicate
TGA	Thermogravimetric analysis
XRD	X-ray diffraction
